# Integrated pro-resolving mechanisms: a new dimension for optimizing anti-inflammatory therapy in asthma – a review of impaired inflammation resolution in airway remodeling

**DOI:** 10.3389/fimmu.2026.1769625

**Published:** 2026-03-23

**Authors:** Bingxue Zhang, Yan Xu, Guihua Song, Mengmeng Sun, Suping Yu, Mingyue Ren

**Affiliations:** 1 Department of Pediatrics, First Affiliated Hospital, Henan University of Chinese Medicine, Zhengzhou, Henan, China; 2 School of Pediatrics, Henan University of Chinese Medicine, Zhengzhou, Henan, China

**Keywords:** airway remodeling, asthma, efferocytosis, eosinophils, inflammation resolution, lipoxins, macrophages, resolvins

## Abstract

The core pathology of asthma involves not only inflammation amplification but also a systemic failure of inflammation resolution programs. This review first summarizes the Specialized Pro-resolving Mediators (SPM) family, efferocytosis, and their molecular basis in maintaining respiratory system homeostasis, emphasizing the positive feedback loop between lipid mediator class switching and macrophage reprogramming. Subsequently, it delineates the structural and functional characteristics of asthmatic airway remodeling and compiles evidence from human samples, animal models, and *in vitro* experiments regarding impaired SPM generation, attenuated receptor signaling, and reduced efferocytosis efficiency. This reveals a cascade mechanism of “SPM deficiency-–efferocytosis impairment-–secondary necrosis-–structural damage.” Furthermore, the review discusses the links between severe/refractory phenotypes, early-onset airway remodeling, and high eosinophilic/mixed granulocytic inflammation with resolution defects from the perspective of clinical phenotypes and biomarkers. It proposes the potential of composite indicators such as SPM profiles, Damage-Associated Molecular Patterns (DAMPs) levels, and phagocytic indices in stratified management and efficacy prediction. In the therapeutic and translational section, the review systematically compares the effects of exogenous SPMs, SPM generation/receptor modulators, efferocytosis-enhancing strategies, and traditional Inhaled Corticosteroids (ICS) and biologics on resolution pathways. It proposes an approach where “pro-resolving pharmacology” synergizes with existing anti-inflammatory therapies. Finally, the review addresses the limitations of current evidence regarding sample size, detection methods, and model constraints, calling for the inclusion of standardized resolution indicators and imaging/histological endpoints in prospective cohorts and clinical trials to promote a shift from symptom control towards structure-oriented, disease-modifying comprehensive interventions.

## 1 Introduction

“Why do some asthma patients continue to experience lung function decline despite high-intensity anti-inflammatory treatment?” This question permeates modern respiratory medicine practice. Bronchial asthma is a highly heterogeneous chronic inflammatory airway disease that has become a persistent global public health challenge. Global Burden of Disease data from 1990–2019 indicate that the incidence and prevalence continue to rise in multiple regions. Although mortality has decreased, the years of life lost due to premature death remain substantial, suggesting that socioeconomic pressures have not been truly alleviated (1). Environmental exposures and structural inequalities further amplify this burden: early-life exposure to traffic-related nitrogen oxides significantly increases the lifelong asthma risk in minority children (2), and asthma-related deaths among adolescents in the United States remain concentrated in medically underserved populations (3). At the therapeutic level, standard regimens (including Inhaled Corticosteroids (ICS), long-acting β2-agonists, and biologics targeting IL-5, IL-4/13, or IgE) can alleviate symptoms and reduce acute exacerbations, but their intervention on “airway remodeling,” a long-term outcome, is very limited. Clinical studies show that even under high-dose ICS control, some children still exhibit severe deficiencies in lipoxin A4 (LXA4) and its receptor Formyl Peptide Receptor 2/ALX (FPR2/ALX). This attenuated endogenous pro-resolving signal is directly associated with ICS resistance and frequent exacerbations (4).

Furthermore, while mepolizumab-induced oral corticosteroid reduction in patients with severe eosinophilic asthma reduced acute exacerbations, it did not eradicate airway structural damage, suggesting that traditional “anti-inflammatory” strategies struggle to reverse the disease course (5). Bronchoalveolar lavage fluid studies further indicate that patients with severe asthma exhibit a “low-low-high” phenotype of lipoxin and resolvin deficiency coupled with elevated serum amyloid A, and these “resolution indicators” are closely associated with clinical outcomes such as acute exacerbations and lung function decline (6). These observations point to a common issue: inflammation suppression does not necessarily mean that inflammation has been healthily terminated (resolved).

Based on this, the scientific community’s understanding of the inflammatory process is shifting from a purely passive suppression to an integrated paradigm of “suppression + active termination.” Resolution of inflammation is considered a dynamic process coordinated by specific lipid mediators and cellular programs, tasked with terminating inflammatory responses, clearing apoptotic cells, and restoring homeostasis (7). This concept has given rise to “pro-resolution pharmacology,” which emphasizes leveraging specific Specialized Pro-resolving Mediators (SPMs) and their receptor networks to complement classic anti-inflammatory treatments and collectively achieve disease modification (8). Notably, similar “resolution impairments” have been described in atherosclerosis, rheumatoid arthritis, and Chronic Obstructive Pulmonary Disease (COPD), suggesting a potential cross-disease pathway of inflammatory homeostatic disruption (7).

Therefore, this review aims to systematically delineate the abnormalities of SPMs and efferocytosis in asthma, analyze their evidence chain with airway remodeling, phenotypic stratification, and treatment response, and explore translational approaches centered on resolution pathways that synergize with traditional anti-inflammatory strategies. To ensure systematic and actionable discussion, this article follows a “mechanism-phenotype-intervention-evidence chain” logic: first, it outlines the biological basis of inflammation resolution pathways; second, it focuses on abnormal manifestations in asthmatic airways and clinical phenotypes; third, it further discusses resolution mechanism-based therapeutic strategies, real-world obstacles, and evidence gaps; finally, it proposes testable future research hypotheses and cross-disease implications.

**Figure 1:**
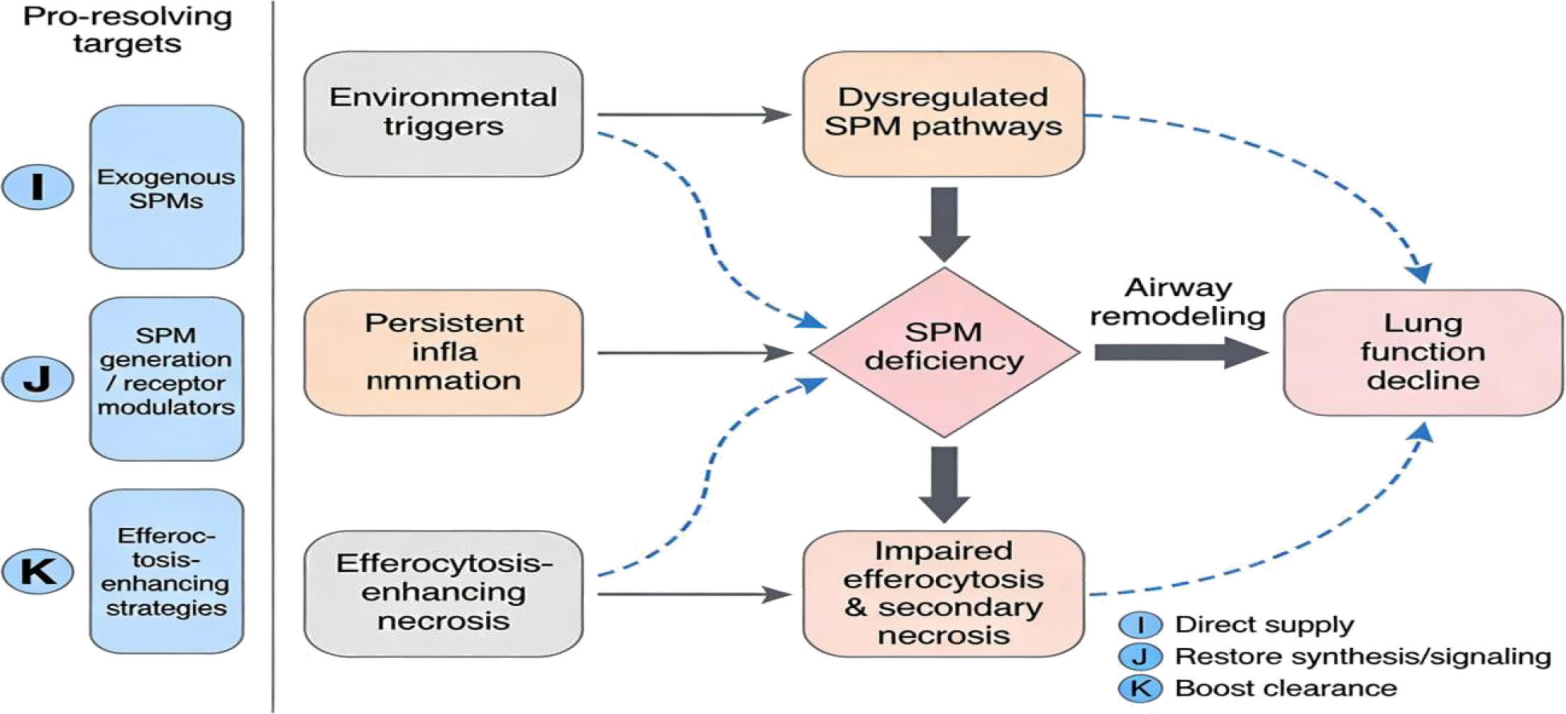
Flowchart diagram illustrating how environmental triggers,persistent inflammation, and impaired efferocytosis contribute to specialized proresolving mediator (SPM) deficiency, leading to airway remodeling and lung function decline, with potential interventions labeled I for direct supply, J for restoring synthesis or signaling, and K for boosting clearance.

**Figure 1** provides a visual overview of this logical chain, facilitating a quick understanding of the key points in subsequent sections. In the legend, intervention nodes I, J, and K represent three complementary pro-resolving strategies that will be discussed in detail in Section 5.

### 1.1 Literature search and selection strategy

This narrative review was conducted by systematically searching PubMed, Web of Science, and Scopus databases (through January 2026) using the following keyword combinations: (‘asthma’ OR ‘airway remodeling’) AND (‘specialized pro-resolving mediators’ OR ‘lipoxins’ OR ‘resolvins’ OR ‘efferocyto sis’ OR ‘inflammation resolution’). We prioritized original research articles, systematic reviews, and meta-analyses published in peer-reviewed journals. Studies were selected based on relevance to the core themes—SPM biology, efferocytosis mechanisms, asthma phenotypes, and therapeutic implications—with preference given to human cohort studies, mechanistic animal models, and clinical trials.

Non-English articles and case reports were excluded unless they provided unique mechanistic insights.

## 2 Biological basis of inflammation resolution pathways

### 2.1 Distinction between inflammation resolution and traditional anti-inflammatory responses

Successful resolution of inflammation is fundamental to maintaining tissue homeostasis and health. Conceptually, inflammation resolution differs fundamentally from traditional anti-inflammatory actions. The core of anti-inflammatory strategies lies in “suppression,” with the primary goal of blocking or attenuating the initiation and amplification signals of inflammation. In contrast, pro-resolving strategies focus on restoring homeostasis by regulating immune cells through specific mediators to terminate inflammation and initiate repair (9).

### 2.2 Efferocytosis and macrophage reprogramming

Efferocytosis is a crucial step in inflammation resolution, specifically referring to the biological process by which phagocytes (primarily macrophages) recognize, engulf, and clear apoptotic cells. This process plays an irreplaceable role in maintaining tissue homeostasis, preventing chronic inflammation, and initiating tissue repair (10). During apoptosis, dying cells actively expose “eat-me” signals, most notably phosphatidylserine (PS) translocation to the outer cell membrane. Phagocytes recognize these signals through surface receptors including tyrosine kinase receptors (MerTK, Axl) and scavenger receptors (CD36, SR-A), as well as bridging molecules like MFG-E8 and Gas6 (11).

Efferocytosis extends beyond debris clearance to exert profound immunomodulatory effects. First, it prevents secondary necrosis by safely disposing of apoptotic cells before membrane integrity is lost. Failure of timely clearance leads to release of DAMPs (HMGB1, ATP, DNA) that re-initiate inflammatory responses (12). Second, efferocytosis induces macrophage phenotypic reprogramming from pro-inflammatory (M1-like) to pro-resolving (M2-like) states. Reprogrammed macrophages secrete anti-inflammatory factors (TGF-β, IL-10) and SPMs while exhibiting enhanced phagocytic capacity (13). This reprogramming involves metabolic shifts including upregulated fatty acid oxidation that determines SPM biosynthesis capacity, creating a positive feedback loop between SPM generation, efferocytosis, and tissue regeneration (14).

### 2.3 Specialized pro-resolving lipid mediators family

Specialized Pro-resolving Mediators (SPMs) are core molecular effectors in the process of inflammation resolution. They are a class of structurally diverse but functionally synergistic endogenous lipid signaling molecules, primarily generated from polyunsaturated fatty acids (PUFAs) through a series of enzymatic reactions. Based on their chemical structure and biosynthetic origin, SPMs are mainly divided into four core families: (1) Lipoxins, derived from arachidonic acid (AA), such as lipoxin A4 (LXA4); (2) E-series resolvins, derived from eicosapentaenoic acid (EPA), such as RvE1 and RvE2; (3) D-series resolvins, Protectins (e.g., neuroprotectin), and maresins, derived from docosahexaenoic acid (DHA), with representative molecules being RvD1, PD1, and MaR1, respectively.

The biosynthesis of SPMs is a precisely regulated process, often involving collaboration between multiple cell types. Key synthetic enzymes include lipoxygenases (LOX), such as 5-LOX, 12-LOX, and 15-LOX, as well as cyclooxygenase-2 (COX-2). These enzymes are induced in inflammatory environments, catalyzing the oxidation and rearrangement of PUFAs to form SPM molecules with specific stereochemical configurations. Once generated, SPMs exert their biological effects by binding to specific G protein-coupled receptors (GPCRs) on the cell surface. These receptors include ALX/FPR2 (lipoxin A4 receptor), ChemR23/CMKLR1 (one of the RvE1 receptors), and GPR32 (one of the RvD1 receptors), among others.

Despite their structural diversity, SPMs share a high degree of functional commonality, collectively forming the “molecular brakes” and “repair initiation” system of inflammation resolution. Their core functions primarily encompass three aspects: first, inhibiting the sustained recruitment and infiltration of inflammatory cells such as neutrophils and eosinophils to the inflammatory site; second, acting as promoters of efferocytosis, enhancing the efficiency of macrophage recognition and clearance of apoptotic inflammatory cells; and third, directly participating in tissue repair and regeneration by regulating fibroblast activity and promoting epithelial cell migration and proliferation (15). These functions collectively maintain tissue homeostasis by remodeling the local inflammatory microenvironment and promoting the shift of immune cells towards a pro-repair phenotype.

**Table 1** Summarizes the major SPM families, their biosynthetic precursors, key receptors, and principal biological functions.

**Table 1:** Summary of specialized pro-resolving mediators (SPMs), their biosynthetic precursors, major receptors, and key biological functions.

SPM family	Precursor	Major receptors	Key functions
Lipoxins (LXA4, LXB4)	Arachidonic acid (AA)	ALX/FPR2	Inhibit neutrophil/eosinophil recruitment; enhance efferocytosis; promote epithelial repair
E-series Resolvins (RvE1, RvE2)	Eicosapentaenoic acid (EPA)	ChemR23, BLT1	Reduce granulocyte infiltration; enhance macrophage phagocytosis; regulate mucus secretion
D-series Resolvins (RvD1-RvD6)	Docosahexaenoic acid (DHA)	GPR32, ALX/FPR2	Suppress inflammatory cytokines; promote M2 macrophage polarization; accelerate tissue regeneration
Protectins (PD1/NPD1)	Docosahexaenoic acid (DHA)	GPR37	Neuroprotection; reduce oxidative stress; enhance resolution in neural and respiratory tissues
Maresins (MaR1, MaR2)	Docosahexaenoic acid (DHA)	LGR6, TRPV1	Enhance efferocytosis; promote tissue repair; regulate pain signaling

### 2.4 General role of resolution pathways in the respiratory system

Inflammation resolution pathways play a pervasive and critical role in the pathophysiology of respiratory diseases. Beyond asthma, SPMs and efferocytosis also influence inflammation persistence and tissue repair in diseases such as pneumonia, Acute Respiratory Distress Syndrome (ARDS), and Chronic Obstructive Pulmonary Disease (COPD), protecting lung structure by limiting granulocyte infiltration, promoting pathogen clearance, and accelerating epithelial repair (16).

The respiratory system, particularly the airways and alveoli, possesses unique barrier structures, mucociliary clearance, and a rich network of immune cells, making the resolution process more complex. Evidence suggests that SPMs not only maintain epithelial barrier integrity and reduce excessive mucus secretion but also promote the regeneration of damaged epithelium and regulate smooth muscle and stromal cell behavior, thereby ensuring that inflammation can be actively terminated and functional homeostasis restored (17).

## 3 Abnormalities in inflammation resolution pathways in asthma

### 3.1 Structural and functional characteristics of airway remodeling

Asthmatic airway remodeling is a significant pathological feature of asthma chronicity, characterized by progressive changes in airway wall structure and irreversible impairment of lung function. These changes not only affect patient symptom control but also predict the long-term prognosis of the disease.

At the structural level, airway remodeling primarily includes the following aspects: (1) epithelial damage and impaired repair; (2) sub-basement membrane fibrosis and collagen deposition; (3) smooth muscle layer thickening and myocyte hypertrophy/hyperplasia; (4) mucous gland and goblet cell hyperplasia (18).

At the functional level, airway remodeling leads to incompletely reversible airway hyperresponsiveness, persistent decline in small airway indices (FEF25-75%, FEV1), and limited peak lung function in childhood-onset patients, indicating that structural abnormalities are embedded in the natural disease course (18). These changes constitute a clinical challenge that existing anti-inflammatory treatments struggle to reverse.

### 3.2 Alterations in SPM generation and signaling in asthma

Abnormal generation and signaling of Specialized Pro-resolving Mediators (SPMs) in the airways of asthma patients are among the key factors contributing to persistent inflammation and airway remodeling. Multiple studies have revealed this defect at various levels. In human samples, SPM levels in asthma patients are generally lower than in healthy controls and are significantly associated with disease severity, lung function parameters, and airway remodeling markers. In bronchoalveolar lavage fluid, induced sputum, and serum, the expression of lipoxin A4 (LXA4) and its receptor Formyl Peptide Receptor 2/ALX (FPR2/ALX) often shows a downward trend, especially in severe or refractory asthma. These defects are closely related to the respiratory inflammatory burden, FEV1 decline, and risk of acute exacerbations (4, 6, 19). Recent lipidomics studies from the U-BIOPRED cohort have identified distinct sputum lipid phenotypes in asthma patients using shotgun mass spectrometry, revealing that phenotypes with higher non-endogenous, cell-derived lipids are associated with significantly worse asthma severity, impaired lung function, and elevated granulocyte counts. Furthermore, omics-based approaches have demonstrated that severe asthma patients exhibit altered lipid metabolic pathways, with reduced pro-resolving lipid mediators correlating with T2-high endotypes and steroid resistance (20).

Abnormal SPM generation and signaling are also reflected in altered expression and function of key metabolic enzymes and receptors. In airway epithelial cells, eosinophils, and macrophages of asthma patients, the expression levels or activities of key SPM synthetic enzymes, such as 15-lipoxygenase (15-LOX), 5-lipoxygenase (5-LOX), and cyclooxygenase-2 (COX-2), may be altered, leading to impaired SPM biosynthesis pathways. Concurrently, the expression of specific SPM receptors, such as lipoxin A4 receptor (ALX/FPR2) and ChemR23, may be downregulated or their signal transduction efficiency reduced in asthmatic airway tissues or inflammatory cells (21). This means that even if small amounts of SPMs are present, their biological effects cannot be fully exerted. Notably, the up-or-down regulation effects of existing drugs (such as Inhaled Corticosteroids (ICS)) on these enzymes and receptors are also being investigated. Some evidence suggests that ICS may exert its anti-inflammatory effects by influencing SPM pathways, but its comprehensive impact on inflammation resolution still requires in-depth exploration (22).

Animal model studies further support the critical role of SPMs in asthma pathophysiology. In allergic airway inflammation models, administration of RvE1, RvD1, or stable LXA4 analogs significantly reduces inflammation, inhibits excessive mucus secretion, decreases airway hyperresponsiveness, and improves airway remodeling indices. Conversely, blocking relevant receptors or reducing their generation exacerbates the pathological phenotype (23–25). It is noteworthy that a “multi-cellular network” comprising airway epithelial cells, granulocytes, and macrophages plays a collaborative role in the SPM pathway: epithelial cells supply precursor fatty acids and express receptors, granulocytes and mast cells provide key enzymes, and macrophages coordinate local balance through receptor signal transduction. The “class synergy” referred to in this article denotes this cross-cellular metabolic-receptor-phagocytic triangular collaboration capacity. Once any node is impaired, it will affect the output of the entire pathway through feedback loops, suggesting that future therapeutic strategies should focus on restoring the synergy of this multi-cellular network rather than single-point regulation (16).

### 3.3 Defective efferocytosis and secondary necrosis

Impaired clearance of apoptotic cells and resulting secondary necrosis in asthmatic airways contribute critically to persistent inflammation and airway remodeling. Secondary necrosis occurs when apoptotic cells lose membrane integrity and release DAMPs (HMGB1, nucleic acids, proteases) that re-activate immune cells, perpetuating chronic inflammation (12). In asthmatic airways, apoptotic eosinophils and neutrophils accumulate abnormally in BALF and induced sputum (26). Macrophages from asthma patients exhibit significantly reduced efferocytosis capacity compared to healthy controls, linked to downregulated phagocytic receptors (MerTK, Axl) (27). When apoptotic eosinophils undergo secondary necrosis, released granule proteins (ECP, MBP, eosinophil peroxidase) directly damage epithelium, disrupt barrier integrity, and stimulate smooth muscle proliferation and fibroblast extracellular matrix production. Clinical studies confirm positive correlations between granule protein levels and disease severity, exacerbation frequency, and remodeling markers (28, 29).

Cross-disease evidence from COPD, interstitial lung disease, and ARDS demonstrates that defective clearance universally drives respiratory tissue damage. This “SPM deficiency-–efferocytosis impairment-–secondary necrosis-–structural damage” cascade provides the framework for biomarkers and therapeutic strategies discussed in subsequent sections (30).

### 3.4 Evidence linking impaired inflammation resolution and airway remodeling

A close association exists between defects in inflammation resolution pathways and asthmatic airway remodeling, manifested not only in abnormalities of Specialized Pro-resolving Mediators (SPMs) generation and efferocytosis but also in how these abnormalities directly or indirectly drive pathological changes in airway structure.

Cross-sectional and cohort studies provide clinical evidence linking SPM levels and macrophage function indicators to the degree of airway remodeling. Multiple studies have found that reduced concentrations of SPMs such as LXA4 and RvD1 in Bronchoalveolar Lavage Fluid (BALF) or induced sputum of asthma patients are negatively correlated with imaging or pathological indicators such as airway wall thickening and sub-basement membrane fibrosis. Concurrently, “resolution imbalance” phenotypes, characterized by decreased macrophage phagocytic capacity and elevated serum amyloid A, are associated with acute exacerbation frequency and lung function decline (31).

It is important to emphasize that most of these studies are cross-sectional, and thus cannot currently establish that “resolution impairment” temporally precedes structural damage; causal inference still requires validation from prospective longitudinal data.

Animal model studies provide more direct mechanistic evidence for this association. As detailed in Section 3.2, allergic airway inflammation models show that genetic knockout or pharmacological inhibition of SPM synthetic enzymes (e.g., 15-lipoxygenase (15-LOX)) or receptors (e.g., Formyl Peptide Receptor 2/ALX (ALX/FPR2)) leads to exacerbated inflammation and airway smooth muscle thickening. Conversely, administration of RvE1, RvD1, or stable LXA4 analogs can reduce airway hyperresponsiveness, inhibit mucous gland hyperplasia, and decrease collagen deposition. Similarly, building on the efferocytosis defects described in Section 3.3, mice lacking efferocytosis receptors such as MerTK struggle to clear eosinophils, exhibiting a more severe remodeling phenotype (16, 31).

Furthermore, the impact of existing anti-inflammatory treatments on resolution pathways offers new perspectives for understanding the link between impaired inflammation resolution and airway remodeling. Some studies suggest that traditional anti-inflammatory drugs like Inhaled Corticosteroids (ICS), in addition to directly suppressing pro-inflammatory responses, may also indirectly promote inflammation resolution by upregulating SPM generation or enhancing macrophage phagocytic function. However, this effect may be insufficient to fully correct inherent resolution defects in asthma, especially in patients with severe or refractory asthma. This explains why some patients experience a decrease in inflammatory markers under ICS treatment, yet airway remodeling continues to progress. Biologics targeting cytokines such as IL-5, IL-4/IL-13, while effectively controlling type 2 inflammation, still require in-depth research into their comprehensive impact on SPM generation and efferocytosis to assess their potential role in preventing airway remodeling (32, 33).

**Table 2** provides a comparative summary of key evidence from animal models and human studies regarding impaired inflammation resolution and airway remodeling in asthma (18, 40).

**Table 2:** Comparative summary of animal model and human study evidence linking impaired inflammation resolution to asthmatic airway remodeling.

Evidence type	Animal model findings	Human study findings	Limitations
SPM Deficiency and Remodeling	OVA/HDM models: 15-LOX knockout mice show increased airway smooth muscle thickening, collagen deposition, and AHR (34)	Severe asthma patients: Reduced BALF/sputum LXA4 and RvD1 correlate with airway wall thickness on CT imaging (31, 35)	Human studies mostly cross-sectional; cannot establish temporal causality
Exogenous SPM Administration	RvE1/RvD1/LXA4 analogs reduce eosinophil infiltration, mucus hypersecretion, and remodeling indices in allergic models (24, 25).	Phase I/II trials ongoing; early data suggest tolerability and multi-target effects	Long-term efficacy and optimal delivery routes not yet established
Efferocytosis Impairment	MerTK-deficient mice show impaired eosinophil clearance, increased secondary necrosis, and exacerbated fibrosis (36).	Asthma patients: Reduced macrophage phagocytic index correlates with elevated eosinophil granule proteins and HMGB1 (37).	*In vitro* assays may not fully reflect *in vivo* complexity
Receptor Signaling Defects	ALX/FPR2 knockout mice exhibit persistent neutrophilic inflammation and structural damage despite steroid treatment	Severe asthma: Downregulated ALX/FPR2 and ChemR23 expression associated with ICS resistance (31).	Receptor polymorphisms and posttranslational modifications understudied
ICS and Biologic Effects	ICS upregulate 15-LOX in some models but effects limited in chronic inflammation contexts	Some ICS-treated children still show LXA4/FPR2 deficiency despite symptom control. Mepolizumab reduces exacerbations but structural damage persists (38, 39).	Species differences (mouse *vs* human) in lipid metabolism; biologic effects on SPM pathways incompletely characterized

In summary, the impaired inflammation resolution pathway and airway structural.abnormalities form a robust multi-evidence chain: biomarker associations revealed by clinical cohorts, causal inferences provided by animal experiments, and partial regulation of pathways by existing therapies collectively establish a causal path of “resolution impairment-–remodeling-–functional decline,” providing a framework for identifying specific phenotypes discussed in the next section.

## 4 Clinical phenotypes and biomarkers

### 4.1 Asthma phenotypes associated with impaired inflammation resolution

Impaired inflammation resolution exhibits high heterogeneity in the clinical manifestations of asthma and is closely associated with specific asthma phenotypes. Understanding these associations can help more precisely identify high-risk patients and formulate individualized treatment strategies.

Patients with severe and refractory asthma often exhibit poor response to traditional anti-inflammatory treatments, with persistent inflammation and progressive airway remodeling tightly linked to resolution defects. These patients show reduced SPM generation and receptor signaling plus low efferocytosis efficiency, leading to apoptotic cell retention that continuously releases DAMPs and exacerbates airway damage (38). Early-onset and persistent airway remodeling phenotypes associate with impaired inflammation resolution. In childhood-onset disease, insufficient early resolution mechanisms lead to abnormal airway structural changes during development, resulting in limited peak lung function in adulthood and increased susceptibility to subsequent inflammatory stimuli (41).

Furthermore, asthma inflammatory phenotypes, particularly high eosinophilic and mixed granulocytic inflammation, have a complex relationship with SPM and efferocytosis indicators. Patients with high eosinophilic asthma often present with lipoxin deficiency and impaired macrophage phagocytosis, leading to granule protein release and structural damage (31).

Mixed granulocytic asthma involves the retention of neutrophils and their Neutrophil Extracellular Traps (NETs), suggesting “clearance defects” in multiple cell lineages (42). In patients with type 2-low asthma or those with obesity, insulin resistance, oxidative stress, or mechanical stretch may drive remodeling through independent pathways, meaning that resolution impairment may be only one component of a multi-factorial network (43). In-depth research into SPM/efferocytosis characteristics in different inflammatory and metabolic phenotypes will help elucidate the relative weight of these mechanisms in asthma heterogeneity and provide a basis for developing precision treatments based on pro-resolving strategies.

In conclusion, phenotypic stratification must incorporate the dimension of “resolution capacity” to explain why some patients respond poorly to standard anti-inflammatory treatments and to identify populations most likely to benefit from targeted pro-resolving therapies.

### 4.2 Potential biomarkers

Given the critical role of impaired inflammation resolution in asthma pathophysiology, identifying and validating relevant biomarkers is of significant importance for early diagnosis, risk stratification, prediction of treatment response, and new drug development. These potential biomarkers can be evaluated across multiple levels of Specialized Pro-resolving Mediator (SPM) pathways, efferocytosis, and inflammatory damage.

First, direct measurement of SPM levels serves as an important potential biomarker. By detecting l (LXA4), RvD, RvE, PD1, MaR, etc. In serum, bronchoalveolar lavage fluid, or induced sputum, inflammation resolution activity can be assessed. Reduced levels of these indicators are associated with disease severity, airway wall thickness, and exacerbation frequency.

Second, key molecules in SPM biosynthesis and signaling (15-lipoxygenase (15-LOX), 5-lipoxygenase (5-LOX), cyclooxygenase-2 (COX-2), and Formyl Peptide Receptor 2/ALX (ALX/FPR2), ChemR23, etc.) in airway tissues, including their expression and activity, can serve as indirect indicators of pathway integrity. Their downregulation is closely associated with poor corticosteroid response (31).

Furthermore, indicators reflecting impaired apoptotic cell clearance are also valuable. Elevated levels of eosinophil granule proteins, Neutrophil Extracellular Traps (NETs), and Damage-Associated Molecular Patterns (DAMPs) (e.g., High-Mobility Group Box-1 (HMGB1)) suggest that apoptotic cells are not cleared promptly, potentially triggering secondary necrosis and inflammation amplification (44). Combining flow cytometry or histological assessment of apoptotic/necrotic cell ratios can more directly reflect the “clearance-death” balance.

Finally, macrophage functional indicators are crucial for assessing efferocytosis efficiency. Assays focusing on macrophage phagocytic index, expression of receptors like MerTK/AXL, and post-phagocytic metabolic reprogramming can reveal whether they maintain a pro-resolving phenotype. In severe asthma, reduced levels of relevant indicators are closely associated with insufficient Prostaglandin E2 (PGE2) and 15-Hydroxyeicosatetraenoic Acid (15-HETE) generation and persistent mucosal damage (45). However, translating these indicators into clinical tools faces multiple challenges: SPM molecules have short half-lives and are extremely sensitive to sampling and storage; mass spectrometry-based detection is costly and lacks standardized quality control across laboratories; and obtaining samples like Bronchoalveolar Lavage Fluid (BALF) is invasive, limiting feasibility in children and real-world cohorts (46). Therefore, composite scores are more suitable as research endpoints in the short term, and clinical promotion still requires addressing issues of detection stability, cost, and accessibility.

In practice, a single biomarker often cannot fully reflect the resolution program. An increasing number of studies tend to construct composite scores of “SPM profile + DAMPs + phagocytic index” and interpret their biological significance in conjunction with sampling time (stable phase/acute exacerbation phase). Standardized composite indicators will become key tools for assessing resolution status in future clinical trials and real-world cohorts.

As shown in **Table 3**, resolution biomarkers offer complementary—not redundant—information compared to existing clinical markers. While eosinophil count and FeNO reflect the magnitude of type 2 inflammation, SPM profiles and phagocytic indices reveal whether inflammation can be actively terminated. This distinction is critical for identifying patients at high risk for remodeling despite adequate symptom control.

**Table 3:** Comparative analysis of traditional clinical biomarkers versus resolution-based biomarkers in asthma.

Biomarker	Feasibility	Specificity	Predictive value
Traditional clinical markers			
Blood eosinophils	High: routine testing, low cost, widely available	Moderate: reflects T2 inflammation burden but not resolution capacity	Predicts response to anti-IL-5 biologics; limited for remodeling risk (47, 48).
FeNO	High: non-invasive, point-of-care, standardized	Moderate: indicates airway eosinophilia and steroid responsiveness	Useful for ICS dose adjustment; does not predict structural progression (49).
Total IgE	High: routine blood test, stable analyte	Low: non-specific; elevated in atopy but not resolution defects	Guides anti-IgE therapy; poor correlation with remodeling (50).
Periostin	Moderate: blood-based but not routine; limited availability	Moderate: T2 biomarker, correlates with subepithelial fibrosis	Predicts anti-IL-13 response; unclear role in efferocytosis defects
Resolution-based biomarkers			
SPM profile (LXA4, RvD1, RvE1)	Low: requires mass spectrometry, invasive sampling (BALF/sputum), unstable analytes	High: directly measures resolution capacity; distinguishes pro-resolving *vs*. pro-inflammatory states	Strongly correlates with exacerbation frequency, FEV1 decline, and airway wall thickness (31).
DAMPs (HMGB1, S100A9)	Moderate: blood/sputum-based; some commercial assays available	High: indicates secondary necrosis and clearance failure; differentiates T2-low phenotypes	Elevated in steroid-resistant asthma; predicts persistent inflammation despite treatment (51).
Phagocytic index (efferocytosis assays)	Low: requires ex vivo macrophage isolation and functional assays; labor-intensive	High: directly assesses macrophage clearance capacity; reflects efferocytosis receptor function	Associated with eosinophil granule protein levels and structural damage progression (44).
Receptor expression (ALX/FPR2, MerTK)	Low: requires bronchial biopsy or specialized flow cytometry; not scalable	High: indicates pathway integrity; receptor downregulation linked to ICS resistance	Identifies patients unlikely to benefit from standard therapy; potential theranostic marker (6).

### 4.3 Evidence related to treatment response and prognosis

Inflammation resolution-related indicators show significant potential in predicting asthma treatment response and disease prognosis. These indicators can not only help clinicians evaluate the effectiveness of existing treatments but also provide a basis for formulating individualized treatment strategies.

Specialized Pro-resolving Mediator (SPM) or efferocytosis-related indicators are associated with the efficacy of Inhaled Corticosteroids (ICS) and biologics. Even with continuous ICS treatment, some children still exhibit insufficient lipoxin A4 (LXA4) and Formyl Peptide Receptor 2/ALX (FPR2/ALX) levels, a defect closely related to ICS resistance and acute exacerbations (38). Therefore, monitoring SPM profiles or macrophage phagocytic function is expected to become a tool for predicting patient response to ICS or biologics, providing a basis for individualized clinical medication.

Furthermore, inflammation resolution-related indicators are closely associated with the long-term prognosis of asthma. Persistently low SPM levels or impaired efferocytosis efficiency suggest that patients are more prone to acute exacerbations, lung function decline, and progression of airway remodeling. This information can complement traditional lung function and inflammatory indicators to identify high-risk populations requiring more aggressive intervention or pro-resolving therapies (44).

Therefore, making “adequate resolution” a treatment goal as important as symptom control, and guiding stepped therapy through dynamic monitoring of the aforementioned indicators, will be a crucial step towards achieving “disease modification” strategies.

## 5 Therapeutic and translational implications based on inflammation resolution

Asthma treatment is moving from “suppressing inflammation” to “actively resolving and terminating inflammation.” Discoveries surrounding Specialized Pro-resolving Mediators (SPMs) and efferocytosis have gradually formed three complementary translational pathways: directly supplementing missing pro-resolving molecules, restoring endogenous generation and signaling within the body, and enhancing the clearance capacity of apoptotic cells. This section unfolds according to this logic and evaluates the position of existing anti-inflammatory regimens within it.

### 5.1 Exogenous SPMs and their analogs

Based on the critical role of Specialized Pro-resolving Mediators (SPMs) in inflammation resolution and tissue repair, exogenous supplementation of SPMs or their stable analogs has emerged as a new direction for asthma treatment.

In animal models of allergic asthma, exogenous administration of various SPMs, such as E-series resolvin RvE1, D-series resolvin RvD1, and lipoxin A4 (LXA4), has shown significant therapeutic effects. They not only reduce inflammatory cell infiltration and inhibit type 2 cytokines like IL-5/IL-13 but also decrease airway hyperresponsiveness and improve airway remodeling indices, including smooth muscle thickening and mucous gland hyperplasia (52).

Currently, early clinical or preclinical data on exogenous SPMs and their analogs are accumulating. Researchers are exploring routes such as nasal spray, inhalation, or systemic administration to evaluate their safety and pharmacokinetic properties in humans. Existing oral or local delivery experiments suggest that these pro-resolving molecules have good tolerability and multi-target advantages. Although large-scale clinical trials are yet to be conducted, these explorations provide a solid foundation for introducing “pro-resolving pharmacology” into asthma treatment.

However, natural SPMs have characteristics such as short half-lives and susceptibility to oxidative degradation. How to achieve stable delivery, avoid systemic side effects, and determine precise therapeutic windows remain core challenges for clinical translation. Structural analogs, prodrugs, or nanodelivery systems developed to address these issues need to provide pharmacokinetic and pharmacodynamic evidence in future Phase I/II trials (53).

### 5.2 Drugs regulating SPM generation and signaling pathways

Pharmacological regulation targeting Specialized Pro-resolving Mediator (SPM) generation and signaling pathways is another important direction for developing novel asthma therapies based on inflammation resolution mechanisms. Inhaled Corticosteroids (ICS) and other traditional drugs can enhance 15-lipoxygenase (15-LOX) and Formyl Peptide Receptor 2 (FPR2) expression in some patients, indirectly promoting lipoxin A4 (LXA4) generation, but the magnitude of this regulation is limited in severe asthma, suggesting a need for more specific “pathway strengthening” strategies (31). SPMs exert their effects by binding to specific G protein-coupled receptors (e.g., Formyl Peptide Receptor 2/ALX (ALX/FPR2), ChemR23). Therefore, developing receptor agonists or homeostatic modulators becomes a viable approach. Existing research shows that molecules targeting ALX/FPR2 can inhibit granulocyte recruitment, enhance efferocytosis, and regulate the pro-inflammatory activity of Natural Killer (NK) cells and Type 2 Innate Lymphoid Cells (ILC2s) (15). Concurrently, stable LXA4 or RvE1 analogs are also being explored for “endogenous amplification” strategies, aiming to replicate multi-target effects in different cell populations (54).

Currently, these modulators are still in preclinical stages. Future research needs to elucidate their pharmacodynamics in different asthma phenotypes, optimize delivery methods, and differentiate them from traditional immunosuppressive treatments to ensure they truly achieve “pro-resolution” rather than simply “anti-inflammation”.

### 5.3 Therapeutic strategies to enhance efferocytosis

Enhancing efferocytosis is another important strategy for treating asthma based on inflammation resolution mechanisms. By promoting the effective clearance of apoptotic cells by macrophages, secondary necrosis-induced inflammation amplification can be avoided, and tissue repair can be promoted (36).

Currently, researchers are exploring various targets to promote efferocytosis. These include upregulating the expression of phagocytic receptors (e.g., MerTK, Axl), increasing the levels of bridging molecules (e.g., MFG-E8, Gas6), or using drugs to directly activate phagocytic signaling pathways. Some studies have shown that drugs like statins and glucocorticoids can, to some extent, enhance efferocytosis, but their mechanisms of action and effects require further clarification (44).

In addition, targeted enhancement of macrophage metabolic reprogramming to maintain their pro-resolving phenotype is also an emerging research direction. By regulating metabolic pathways such as fatty acid oxidation and mitochondrial function, macrophage phagocytic capacity and SPM generation capacity can be enhanced (55). Although these strategies are still in early research stages, they provide new ideas for reversing persistent inflammation and airway remodeling in asthma.

### 5.4 Effects of existing anti-inflammatory drugs on inflammation resolution

Traditional asthma anti-inflammatory treatments, such as Inhaled Corticosteroids (ICS) and biologics, may have dual roles in inflammation resolution, both promoting and limiting certain resolution processes.ICS may indirectly promote inflammation resolution by reducing the inflammatory burden and upregulating SPM synthetic enzymes and receptors to some extent. However, its comprehensive impact on SPM generation and efferocytosis still requires in-depth research. In some patients, especially those with severe or refractory asthma, the promoting effect of ICS on inflammation resolution may be insufficient, leading to persistent inflammation and airway remodeling despite treatment. Biologics targeting type 2 cytokines (e.g., anti-IL-5, anti-IL-4/13, anti-IgE) can effectively control type 2 inflammation and reduce inflammatory cell infiltration and cytokine levels. Their comprehensive impact on SPM generation and efferocytosis has not been fully elucidated. Some studies suggest that these biologics may indirectly promote inflammation resolution by reducing the inflammatory burden, but whether they can directly enhance SPM generation or efferocytosis still requires further investigation (44).Therefore, combining traditional anti-inflammatory drugs with pro-resolving strategies may provide more comprehensive disease control and even achieve “disease modification” goals.

### 5.5 Rationale for combined therapies

Given that asthma pathophysiology involves both inflammatory amplification and resolution impairment, a treatment strategy combining “suppression” and “active termination” has theoretical advantages.

On one hand, traditional anti-inflammatory drugs (e.g., ICS, biologics) can quickly control acute inflammation and reduce inflammatory cell infiltration and cytokine release. On the other hand, pro-resolving therapies (e.g., exogenous SPMs, efferocytosis enhancers) can promote the healthy termination of inflammation and tissue repair, avoiding chronic inflammation and structural damage caused by resolution defects (13).

This combined approach is expected to achieve more comprehensive disease control, reduce relapse rates, and potentially reverse airway remodeling to some extent. Although this concept is highly promising, its clinical application still requires validation through well-designed clinical trials.

## 6 Evidence gaps and future research directions

### 6.1 Limitations at the evidence level

Although existing research has revealed the critical role of impaired inflammation resolution in asthma, there are still significant limitations in current evidence.

First, most clinical studies are cross-sectional or small-sample studies, unable to establish causal relationships between resolution defects and airway remodeling or long-term prognosis. Prospective, large-sample, multi-center cohort studies are needed to clarify the temporal relationship between resolution pathway abnormalities and disease progression (44).

Second, current detection methods for SPMs and efferocytosis-related indicators still face many technical challenges. SPM molecules have short half-lives and are unstable, and mass spectrometry-based detection methods are costly and lack standardized quality control across laboratories. Efferocytosis functional assays require specialized techniques and are difficult to implement widely in clinical settings. Developing simple, rapid, stable, and cost-effective detection methods is key to translating resolution biomarkers into clinical applications (13).

Third, animal model studies, while providing important mechanistic insights, have limitations in reproducing the complexity and heterogeneity of human asthma. For example, there are significant differences in lipid metabolism pathways between mice and humans, and some SPM receptors have different expression patterns and functions across species. Therefore, caution is required when extrapolating animal model findings to human clinical practice.

Fourth, current research on pro-resolving therapies is mostly in preclinical or early clinical stages, lacking large-scale, randomized controlled trials. The safety, efficacy, optimal dosage, and delivery methods of these therapies require further investigation. In particular, the effects of exogenous SPMs or efferocytosis enhancers in different asthma phenotypes and whether they can truly achieve “disease modification” goals need validation through long-term follow-up studies (56).

### 6.2 Priorities for future research

Based on current evidence gaps, future research should focus on the following areas:

First, conduct prospective, large-sample, multi-center cohort studies to systematically assess the predictive value of resolution-related biomarkers (SPM profiles, DAMPs, phagocytic indices) for asthma exacerbations, lung function decline, and airway remodeling progression. These studies should include standardized SPM detection platforms, longitudinal sampling strategies, and comprehensive clinical phenotyping.

Second, develop and validate simple, rapid, stable, and cost-effective resolution biomarker detection methods suitable for clinical application. This may include point-of-care testing devices, stable SPM analogs as surrogate markers, or composite scoring systems based on readily available clinical parameters.

Third, conduct in-depth mechanistic research on the comprehensive impact of existing anti-inflammatory drugs (ICS, biologics) on resolution pathways using advanced omics technologies (transcriptomics, proteomics, lipidomics, metabolomics). This will help optimize existing treatment regimens and identify patient subgroups most likely to benefit from pro-resolving therapies (57).

Fourth, advance clinical translation of pro-resolving therapies through well-designed Phase II/III randomized controlled trials. These trials should assess the safety and efficacy of exogenous SPMs, SPM receptor agonists, and efferocytosis enhancers in different asthma phenotypes, with endpoints including not only symptom control and exacerbation frequency but also structural remodeling indicators (e.g., airway wall thickness on CT imaging, subepithelial fibrosis on bronchial biopsy).

Fifth, explore the role of resolution pathways in asthma phenotype stratification and precision medicine. Identifying specific “resolution-deficient” phenotypes and developing targeted pro-resolving therapies for these populations may represent an important direction for future precision asthma treatment (58).

Finally, investigate cross-disease commonalities and differences in resolution pathway abnormalities. Since similar resolution defects have been described in diseases such as COPD, atherosclerosis, and rheumatoid arthritis, comparative studies across diseases may reveal common therapeutic targets and provide insights for drug repurposing (59).

### 6.3 Clinical translation challenges

Translating pro-resolving therapies from bench to bedside faces multiple practical challenges beyond scientific evidence.

First, regulatory approval pathways for pro-resolving therapies are unclear. Since SPMs are endogenous molecules rather than traditional pharmaceuticals, their regulatory classification (drug, biological product, or nutritional supplement) and approval requirements need clarification from regulatory agencies.

Second, manufacturing and quality control of SPM-based therapeutics pose technical challenges. Ensuring batch-to-batch consistency, stability during storage and transportation, and cost-effectiveness at scale require advanced pharmaceutical development.

Third, reimbursement and accessibility issues may limit clinical adoption. Without clear evidence of cost-effectiveness compared to existing therapies, health insurance systems may be reluctant to cover expensive SPM-based treatments or specialized resolution biomarker testing.

Fourth, physician education and clinical practice integration are essential. Most clinicians are unfamiliar with resolution biology concepts and may require training to appropriately identify candidates for pro-resolving therapies and interpret resolution biomarkers.

Addressing these challenges will require collaborative efforts among researchers, pharmaceutical companies, regulatory agencies, payers, and clinical practitioners.

## 7 Conclusions

Asthma is not merely a disease of excessive inflammation but also reflects a fundamental failure of inflammation resolution programs. Impaired generation and signaling of Specialized Pro-resolving Mediators (SPMs), coupled with defective efferocytosis, create a cascade of “SPM deficiency-–efferocytosis impairment-–secondary necrosis-–structural damage” that drives airway remodeling and disease persistence. Emerging evidence from human cohorts, animal models, and *in vitro* studies establishes resolution defects as key determinants of severe/refractory phenotypes, early-onset remodeling, and poor treatment responses.

Resolution-based biomarkers (SPM profiles, DAMPs, phagocytic indices) offer complementary information to traditional inflammatory markers, uniquely capturing the active termination capacity of inflammation. Their integration into clinical phenotyping may enable more precise risk stratification and treatment selection. Therapeutically, pro-resolving strategies—including exogenous SPMs, SPM pathway modulators, and efferocytosis enhancers—represent promising complements to traditional anti-inflammatory therapies, with potential to achieve true disease modification rather than mere symptom suppression.

However, significant evidence gaps remain. Most clinical studies are cross-sectional; biomarker detection methods lack standardization and scalability; and pro-resolving therapies await validation in large randomized trials. Future research must prioritize prospective cohorts with standardized resolution endpoints, develop clinically feasible biomarker platforms, elucidate the impact of existing drugs on resolution pathways through omics approaches, and advance clinical translation through well-designed trials incorporating structural remodeling endpoints.

The conceptual shift from “suppressing inflammation” to “actively resolving inflammation” has profound implications for asthma management. By integrating resolution biology into clinical practice, we move closer to a paradigm where treatment goals extend beyond symptom control to encompass structural preservation and disease course modification. This represents not an abandonment of existing anti-inflammatory strategies but their evolution into a more comprehensive, biologically informed approach that respects the active, programmed nature of inflammation termination. The resolution pathway, once a neglected dimension of asthma pathophysiology, now emerges as a critical frontier for precision medicine and therapeutic innovation.
